# Thyroid-Liver Axis: Mechanistic Insights and Clinical Implications

**DOI:** 10.7759/cureus.110637

**Published:** 2026-06-11

**Authors:** Naima Parveen, Sachin Chittawar, Deepak Khandelwal, Sameer Aggarwal

**Affiliations:** 1 Research, Harmony - Dr Sachin's 360 Degree Diabetes Care, Bhopal, IND; 2 Endocrinology, Harmony - Dr Sachin's 360 Degree Diabetes Care, Bhopal, IND; 3 Endocrinology and Diabetes, Khandelwal Diabetes, Thyroid and Endocrinology Clinic, Delhi, IND; 4 Endocrinology, Dr Sameer Aggarwal Endocrine Centre, Rohtak, IND

**Keywords:** hyperthyroidism, hypothyroidism, liver, metabolic dysfunction-associated steatotic liver disease, thyroid hormone

## Abstract

The thyroid gland and liver share a complex bidirectional relationship that is fundamental to metabolic regulation and hormonal homeostasis. Thyroid hormones (THs) regulate hepatic lipid handling, glucose metabolism, mitochondrial function, and energy balance, while the liver governs TH transport, activation, metabolism, and clearance. Increasing evidence links thyroid dysfunction with metabolic dysfunction-associated steatotic liver disease (MASLD), fibrosis progression, and adverse metabolic outcomes. This narrative review provides an updated synthesis of the mechanistic, clinical, and therapeutic aspects of thyroid-liver interactions and their implications for clinical practice. A comprehensive review of mechanistic, clinical, translational, and therapeutic studies examining thyroid dysfunction, liver disease, thyroid hormone sensitivity, and emerging thyroid hormone-based therapies was conducted. Thyroid dysfunction contributes to MASLD, dyslipidemia, insulin resistance, and hepatic injury, whereas liver disease alters TH metabolism and complicates the interpretation of thyroid function tests, including the occurrence of nonthyroidal illness syndrome. Emerging evidence suggests that reduced intrahepatic TH signaling and altered tissue-level hormone sensitivity play central roles in steatosis and fibrosis progression. Clinically, recognition of these interactions may improve the interpretation of thyroid abnormalities in liver disease and support risk stratification in metabolic liver disorders. The development of liver-targeted thyroid hormone receptor-β agonists, including resmetirom, represents a major therapeutic advance with potential to reshape management strategies for metabolic dysfunction-associated steatohepatitis (MASH). However, important controversies remain regarding the diagnostic utility of thyroid hormone sensitivity indices, long-term safety of thyromimetics, and the role of thyroid hormone replacement in liver-directed therapy, highlighting the need for robust prospective studies.

## Introduction and background

The thyroid gland and the liver are deeply interconnected organs that together regulate systemic energy balance, lipid handling, glucose metabolism, and hormonal homeostasis. Thyroid hormones (THs) influence virtually every aspect of hepatic metabolism, while the liver plays a central role in thyroid hormone (TH) transport, peripheral activation, degradation, and excretion. Through these reciprocal interactions, disturbances in either organ can propagate metabolic dysfunction across multiple physiological systems [1,2].

TH, particularly triiodothyronine (T3), exerts direct genomic and non-genomic effects on the hepatocytes, regulating pathways involved in lipid oxidation, cholesterol synthesis, gluconeogenesis, and protein turnover. These effects are mediated predominantly through thyroid hormone receptor beta (TRβ), the principal receptor isoform expressed in the liver, and are finely tuned by intracellular deiodinase activity and membrane transporters that determine local hormone availability [1,3]. Conversely, the liver synthesizes TH-binding proteins, including thyroxine-binding globulin (TBG), transthyretin, and albumin, and is the primary site for conjugation and biliary excretion of THs, thereby influencing circulating hormone concentrations and bioactivity [1,3].

Both hypothyroidism and hyperthyroidism are associated with clinically significant hepatic manifestations. Hypothyroidism is frequently accompanied by dyslipidemia, reduced hepatic fatty acid oxidation, insulin resistance, and an increased prevalence of metabolic dysfunction-associated steatotic liver disease (MASLD), even in its subclinical form [1,4]. Mild elevations of liver enzymes and cholestatic patterns may also be observed, reflecting impaired bile acid metabolism and reduced hepatic clearance [5]. In contrast, hyperthyroidism is commonly associated with abnormalities in liver function tests (LFT), ranging from mild transaminase elevation to severe hepatocellular injury in the context of thyroid storm or thyrotoxic heart failure. In addition, antithyroid drugs (ATD) may independently contribute to hepatic injury, further complicating clinical assessment [5,6].

Liver disease, in turn, profoundly alters TH metabolism. Acute and chronic hepatic disorders are frequently associated with changes in serum TH profiles, including reduced total and free T3 levels, altered thyroxine (T4) concentrations, and variable thyroid-stimulating hormone (TSH) responses. These changes are driven by reduced synthesis of binding proteins, altered deiodinase expression, impaired cellular uptake of THs, and inflammatory cytokine-mediated suppression of hypothalamic-pituitary-thyroid axis signaling [1,7]. As a result, distinguishing true thyroid dysfunction from adaptive endocrine responses such as nonthyroidal illness syndrome (NTIS) remains a persistent clinical challenge in patients with liver disease [8].

Recent advances have expanded the understanding of thyroid-liver interactions beyond systemic hormone levels. Accumulating experimental and clinical evidence suggests that metabolic liver disease may be characterized by reduced intrahepatic TH signaling despite apparently normal circulating THs. Downregulation of hepatic deiodinase type 1 (DIO1), altered thyroid hormone receptor (TR) expression, and impaired intracellular T3 generation have been implicated in the progression of steatosis, inflammation, and fibrosis [1,9]. These insights have catalyzed the development of liver-targeted TRβ agonists, culminating in the recent approval of resmetirom for the treatment of metabolic dysfunction-associated steatohepatitis (MASH), marking a significant shift in therapeutic strategy [10,11].

Despite these advances, substantial gaps remain in the interpretation and clinical application of thyroid function testing in liver disease. Epidemiological studies report heterogeneous associations between TH levels and hepatic outcomes, influenced by differences in metabolic status, disease definitions, and population characteristics [4]. Emerging concepts such as tissue-specific TH resistance and altered hormone sensitivity challenge the traditional reliance on serum TSH as a surrogate for TH action at the organ level [1,8]. In addition, current evidence remains fragmented across mechanistic, epidemiological, and therapeutic studies, limiting the integration of thyroid-liver interactions into routine clinical practice. Important uncertainties persist regarding the diagnostic interpretation of thyroid function abnormalities in liver disease, the prognostic significance of altered TH signaling, and the long-term safety and efficacy of liver-targeted thyromimetics. Furthermore, existing reviews have often focused on isolated mechanistic or therapeutic aspects rather than providing a unified translational perspective integrating molecular pathways, clinical manifestations, and emerging therapies.

This review integrates mechanistic, clinical, and translational evidence to provide a contemporary synthesis of thyroid-liver interactions. By examining hypothyroidism, hyperthyroidism, TH resistance, and emerging therapeutic approaches within a unified framework, the review aims to clarify unresolved controversies and highlight future directions for research and clinical management in thyroid-related liver diseases.

This review addresses the following focused questions: How does hypothyroidism contribute to the development and progression of MASLD and hepatic fibrosis? How does liver disease alter thyroid hormone metabolism and interpretation of thyroid function tests? What is the clinical significance of nonthyroidal illness syndrome in acute and chronic liver disease? Can indices of thyroid hormone sensitivity improve prognostic assessment beyond conventional thyroid function tests in patients with liver disease? What is the therapeutic potential and safety profile of TRβ selective agonists in MASLD/MASH?

This review is intended for endocrinologists, hepatologists, internists, metabolic disease specialists, and trainees involved in the care of patients with thyroid and liver disorders. The synthesis aims to support clinical decision-making in scenarios such as interpreting thyroid function abnormalities in cirrhosis, evaluating metabolic consequences of thyroid dysfunction, and understanding emerging thyroid hormone-based therapies for MASLD/MASH.

## Review

Search strategy and literature selection

A narrative literature review was conducted using PubMed/MEDLINE, Embase, Scopus, and Google Scholar databases from inception through January 2026. Search terms included combinations of medical subject headings (MeSH) and keywords such as “thyroid hormone,” “thyroid dysfunction,” “hypothyroidism,” “hyperthyroidism,” “liver disease,” “non-alcoholic fatty liver disease,” “NAFLD,” “non-alcoholic steatohepatitis,” “NASH,” “metabolic dysfunction-associated steatotic liver disease,” “MASLD,” “metabolic dysfunction-associated steatohepatitis,” “MASH,” “cirrhosis,” “nonthyroidal illness syndrome,” “thyroid hormone receptor beta agonists,” “resmetirom,” and “thyromimetics.” Boolean operators (AND/OR) were used to refine the search strategy.

Priority was given to high-quality evidence including randomized controlled trials, meta-analyses, systematic reviews, translational studies, and major clinical guidelines published in English. Seminal mechanistic studies and landmark clinical trials relevant to thyroid-liver interactions were also included. Additional references were identified through manual screening of bibliographies from selected articles. The final selection of studies was based on relevance to the mechanistic, clinical, diagnostic, and therapeutic themes addressed in this review.

TH metabolism and signaling in the liver

The liver is a principal site for TH metabolism and action, serving not only as a metabolic target organ but also as a regulator of systemic TH availability. Circulating T4 and T3 enter hepatocytes through specific membrane transporters rather than passive diffusion. Among these, monocarboxylate transporter 8 (MCT8), monocarboxylate transporter 10 (MCT10), and organic anion transporting polypeptides (OATPs) play a critical role in hepatic TH uptake. Alterations in the expression or function of these transporters can significantly influence intracellular TH concentrations, even in the presence of normal circulating levels, thereby contributing to tissue-specific dysregulation [12,13].

Once inside the hepatocyte, THs undergo tightly regulated intracellular activation and inactivation. Although T4 represents the predominant circulating hormone, T3 is the biologically active form that binds nuclear TRs to modulate gene transcription. The conversion of T4 to T3 within the liver is primarily mediated by iodothyronine deiodinase type 1 (DIO1), which is abundantly expressed in hepatocytes and contributes substantially to both local and systemic T3 production. In contrast, deiodinase type 3 (DIO3), which inactivates THs, is minimally expressed in healthy adult liver but may be upregulated in pathological states, leading to reduced intracellular T3 availability [14,15].

The genomic actions of THs in the liver are predominantly mediated through TRβ, the dominant receptor isoform in hepatic tissue. Upon binding T3, TRβ forms heterodimers with retinoid X receptors and interacts with TH response elements on target genes involved in lipid metabolism, cholesterol homeostasis, gluconeogenesis, and mitochondrial function. Through these mechanisms, THs enhance fatty acid β-oxidation, reduce hepatic lipid accumulation, and modulate bile acid synthesis. Disruption of TRβ signaling has been shown to promote steatosis and metabolic dysfunction, underscoring its central role in hepatic metabolic regulation [2,5].

TH signaling within the liver is not uniform across all cell types. While hepatocytes represent the primary site of TH-mediated metabolic effects, non-parenchymal cells, including hepatic stellate cells, Kupffer cells, and sinusoidal endothelial cells, also express components of the TH signaling machinery. Emerging evidence suggests that THs may influence inflammatory responses, fibrogenesis, and vascular tone through actions on these cell populations. For instance, altered TH signaling in hepatic stellate cells has been implicated in fibrotic progression, whereas effects on Kupffer cells may modulate inflammatory cascades relevant to steatohepatitis [16].

**Figure 1:**
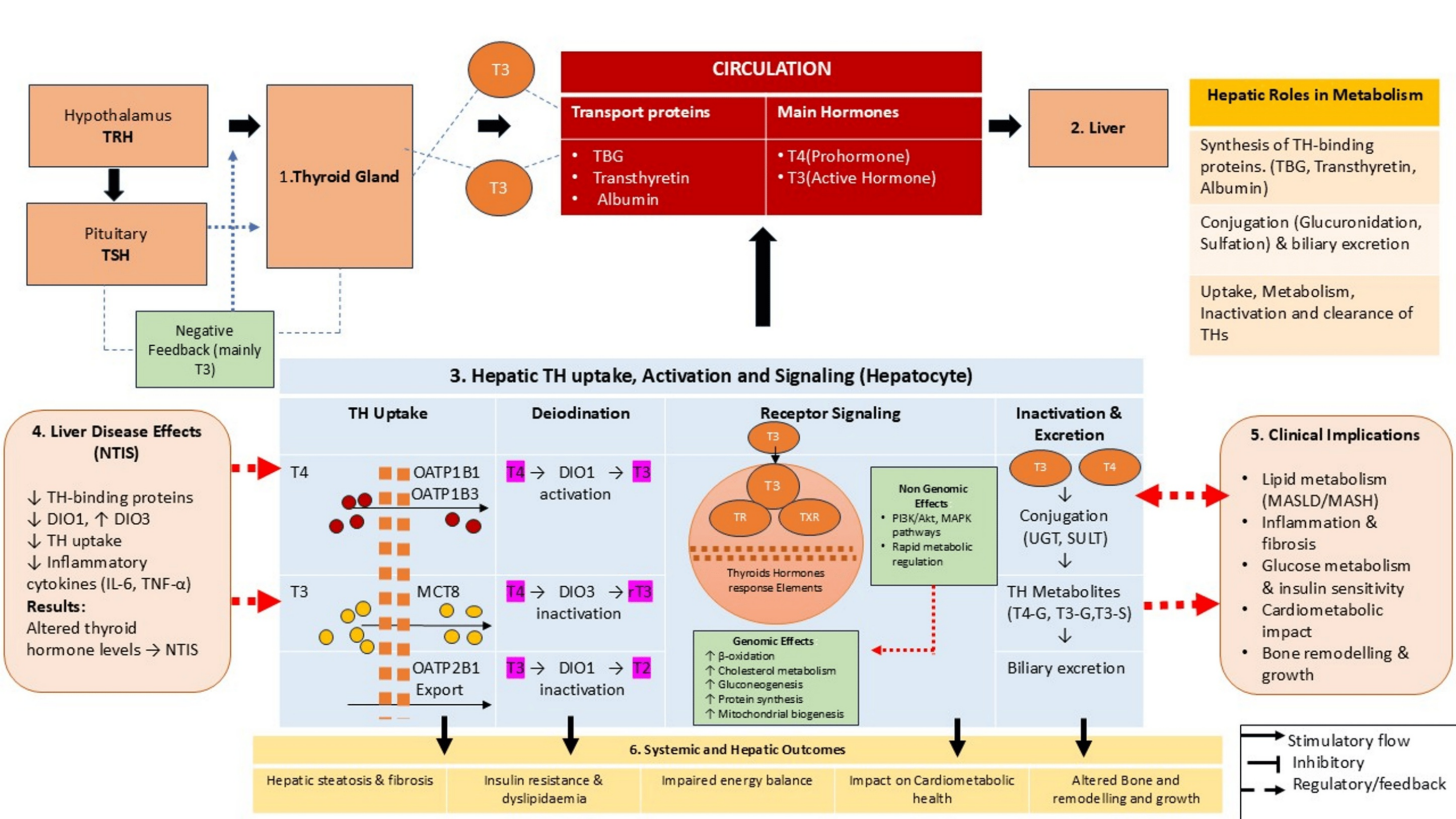
Overview of the thyroid-liver axis and hepatic thyroid hormone signaling TRH: thyrotropin-releasing hormone, TSH: thyroid-stimulating hormone, T4: thyroxine (prohormone), rT3: reverse T3 (inactive), T2: diiodothyronine, TRβ: thyroid hormone receptor beta, RXR: retinoid X receptor, OATP: organic anion transporting polypeptide, MCT8: monocarboxylate transporter 8, DIO1/3: deiodinase type 1, UGT: UDP glucuronosyltransferase, SULT: sulfotransferase, TH-G: glucuronidated TH, TH-S: sulfated TH, NTIS: nonthyroidal illness syndrome, HPT axis: hypothalamic-pituitary-thyroid axis, TBG: thyroxine-binding globulin, MASLD: metabolic dysfunction-associated steatotic liver disease, MASH: metabolic dysfunction-associated steatohepatitis, IL-6: interleukin-6, TNF-α: tumor necrosis factor-alpha, FA: fatty acids, PI3K: phosphatidylinositol 3-kinase, Akt: protein kinase B, cAMP: cyclic adenosine monophosphate, MAPK: mitogen-activated protein kinase, CaMK3: calcium/calmodulin-dependent protein kinase III, HPT: hypothalamic-pituitary-thyroid.

Intrahepatic TH availability is ultimately determined by a complex interplay of systemic hormone supply, hepatic uptake mechanisms, intracellular conversion, receptor expression, and local inflammatory and metabolic milieu. Factors such as insulin resistance, oxidative stress, cytokine signaling, and mitochondrial dysfunction can impair deiodinase activity and receptor responsiveness, leading to reduced effective TH action despite normal circulating TH levels. This concept of dissociation between circulating hormone levels and tissue-level action has gained increasing relevance in metabolic liver disease and provides a mechanistic basis for the emerging notion of intrahepatic hypothyroidism [2,17].Together, these mechanisms highlight the liver as a highly specialized and dynamic organ for TH metabolism and signaling. Understanding the determinants of intrahepatic TH action is essential for interpreting thyroid function abnormalities in liver disease and for the rational development of targeted thyroid-based therapies. The major mechanisms underlying hepatic thyroid hormone transport, metabolism, signaling, and downstream metabolic effects are summarized in Figure 1.

Hypothyroidism and liver function

Hypothyroidism exerts multifaceted effects on liver physiology, reflecting the central role of THs in regulating hepatic energy metabolism, lipid handling, and bile acid homeostasis. Both overt and subclinical hypothyroidism (SCH) have been consistently associated with metabolic derangements that predispose to hepatic dysfunction, even in the absence of primary liver disease. The reduction in TH-mediated metabolic rate leads to impaired mitochondrial β-oxidation of fatty acids, decreased hepatic lipid export, and increased intrahepatic triglyceride accumulation, creating a metabolic milieu favorable for steatosis [1].

One of the most prominent hepatic manifestations of hypothyroidism is dyslipidemia, characterized by elevated total cholesterol and low-density lipoprotein (LDL) cholesterol levels. This lipid profile results from reduced expression of hepatic LDL receptors and diminished activity of cholesterol-catabolizing enzymes, processes that are directly regulated by T3. The consequent lipid overload contributes to hepatic fat accumulation and provides a mechanistic link between hypothyroidism and MASLD. Epidemiological studies have demonstrated a higher prevalence of both overt and SCH in individuals with steatotic liver disease, independent of traditional metabolic risk factors [2,17].

Hypothyroidism is also associated with alterations in liver enzyme profiles. Mild elevations of serum aminotransferases and, less commonly, cholestatic patterns have been reported. These biochemical abnormalities are thought to arise from reduced bile flow, impaired bile acid synthesis, and decreased hepatic clearance of bilirubin and other metabolites [18,19]. Severe and prolonged hypothyroidism may even lead to myxedematous infiltration of hepatic tissue and reduced hepatic blood flow, further exacerbating functional impairment [20].

Beyond steatosis, hypothyroidism may influence the progression of chronic liver disease (CLD) through effects on insulin sensitivity, inflammation, and fibrogenesis. THs exert anti-fibrotic actions by modulating hepatic stellate cell activation and extracellular matrix turnover [16]. Reduced TH signaling has been associated with enhanced fibrogenic pathways and increased susceptibility to progressive liver injury in experimental models [21]. These observations support the concept that hypothyroidism may not only contribute to the development of liver disease but may also influence its natural history and further progression.

TH replacement therapy has been shown to improve lipid profiles and metabolic parameters in overt hypothyroidism, with emerging evidence suggesting potential benefits for hepatic steatosis. Small interventional studies and post hoc analyses indicate that levothyroxine (LT4) therapy may reduce liver fat content and improve aminotransferase levels in selected patients, particularly those with coexisting metabolic dysfunction [9]. Nevertheless, robust randomized controlled trials specifically targeting hepatic outcomes are limited, and concerns regarding overtreatment and cardiovascular risk, especially in older individuals, warrant cautious interpretation [22].

Hyperthyroidism and liver function

Hyperthyroidism is frequently associated with biochemical abnormalities of liver function, reflecting the profound metabolic and hemodynamic effects of excess TH. Mild elevations in serum aminotransferases, alkaline phosphatase (ALP), and bilirubin are commonly observed in untreated thyrotoxicosis and may occur in the absence of underlying liver disease. These abnormalities are often reversible with the restoration of euthyroidism, underscoring a functional rather than structural basis in most cases [6].

Several mechanisms have been proposed to explain hepatic dysfunction in hyperthyroidism. Excess TH increases hepatic oxygen consumption and metabolic demand without a commensurate increase in hepatic blood flow, resulting in relative hypoxia, particularly in the centrilobular regions of the liver. In addition, THs enhance lipolysis and free fatty acid flux to the liver, increasing oxidative stress and mitochondrial dysfunction, which may contribute to hepatocellular injury [1]. In severe cases, these metabolic effects may culminate in cholestasis or acute liver failure, particularly in the context of thyroid storm [6].

Cardiac dysfunction represents an important indirect contributor to liver injury in hyperthyroidism. Thyrotoxicosis-induced high-output heart failure and atrial fibrillation can lead to hepatic congestion and ischemic hepatitis, manifesting as marked elevations in aminotransferases and bilirubin. These changes are more common in older individuals and in those with pre-existing cardiovascular disease, highlighting the interplay between thyroid, cardiac, and hepatic physiology [5].

Drug-induced liver injury is another clinically relevant consideration in hyperthyroid patients. ATD, particularly propylthiouracil, have been associated with severe hepatotoxicity, including fulminant hepatic failure requiring liver transplantation [23]. Methimazole is more commonly associated with cholestatic or mixed patterns of liver injury but is generally considered relatively safer from a hepatic standpoint. These risks have led to changes in clinical practice guidelines for the management of hyperthyroidism, favoring methimazole as first-line therapy in non-pregnant adults and reserving propylthiouracil only for specific indications [24,25].

Hyperthyroidism may exacerbate pre-existing liver disease through increased metabolic demand, enhanced oxidative stress, and alterations in hepatic lipid and bile acid metabolism, thereby worsening hepatic injury in patients with CLD or MASLD [1,26]. Conversely, impaired liver function can complicate the management of hyperthyroidism by altering drug metabolism and increasing susceptibility to ATD-induced hepatotoxicity [27]. Careful interpretation of liver biochemistry and close monitoring during ATD treatment are therefore essential in patients with underlying liver disease [28].

Thyroid function testing and hormone sensitivity in liver disease

Assessment of thyroid function in patients with liver disease presents unique challenges, as conventional serum markers may not accurately reflect tissue-level TH action. TSH has traditionally been used as the primary indicator of thyroid status; however, its interpretation in patients with liver disease is complicated by alterations in hypothalamic-pituitary regulation, changes in TH-binding proteins, and the influence of systemic illness. As a result, reliance on serum TSH alone may lead to misclassification of thyroid status, particularly in patients with CLD or acute hepatic decompensation [2,29].

Free TH measurements provide additional, though still imperfect, insights. Free T3 is frequently reduced in CLD and critical illness, reflecting impaired peripheral conversion of T4 to T3 due to altered deiodinase activity. This pattern, often referred to as low T3 syndrome, has been associated with disease severity and adverse outcomes in cirrhosis and acute liver failure [30]. Free T4 levels may remain normal or be modestly altered, further complicating interpretation when viewed in isolation [29,31]. Consequently, discordant thyroid function test results are common in liver disease and do not necessarily indicate primary thyroid pathology.

Beyond absolute hormone concentrations, increasing attention has been directed toward indices of TH sensitivity and feedback regulation. Derived metrics such as the free T3 to free T4 ratio, the TSH index, and the thyrotroph thyroxine resistance index have been proposed as markers of peripheral and central TH responsiveness. These indices aim to capture variations in TH action that are not apparent from conventional tests and may be particularly relevant in conditions characterized by altered hormone metabolism, including MASLD [32,33].

Interpretation of thyroid function tests is further complicated during acute illness and hepatic decompensation [8]. Inflammatory cytokines, caloric deprivation, and altered hepatic clearance collectively suppress TH activation and pituitary responsiveness, producing biochemical patterns that overlap with SCH or central hypothyroidism [7]. Importantly, these changes are often reversible with clinical recovery and these patients do not consistently benefit from TH replacement, underscoring the need for cautious interpretation and avoidance of unnecessary treatment in acutely ill patients [34,35].

Emerging evidence suggests that TH sensitivity metrics may have a role in risk stratification and prognostication in liver disease. Lower free T3 levels and reduced free T3 to free T4 ratios have been associated with increased mortality, greater fibrosis burden, and poorer clinical outcomes in cirrhosis and metabolic liver disease [36]. Although these indices are not yet incorporated into routine clinical practice, they offer a promising framework for refining endocrine assessment beyond traditional biochemical thresholds and for identifying patients who may benefit from targeted metabolic or thyroid-based interventions [37]. As shown in Table 1, thyroid function test abnormalities in liver disease are heterogeneous and strongly influenced by disease severity. A progressive decline in free T3 levels is evident across compensated and decompensated cirrhosis, reflecting impaired peripheral TH activation rather than primary thyroid dysfunction.

**Table 1:** Typical thyroid function test patterns across liver conditions TSH: thyroid-stimulating hormone; FT4: free thyroxine; FT3: free triiodothyronine; MASLD: metabolic dysfunction-associated steatotic liver disease; MASH: metabolic dysfunction-associated steatohepatitis; TFT: thyroid function test.

Liver condition	TSH	Free T4 (FT4)	Free T3 (FT3)	Common pattern / interpretation	Key references
Metabolic dysfunction-associated steatotic liver disease (MASLD)	Normal to mildly decreased	Normal to mildly increased	Normal to decreased	Reduced peripheral conversion of T4 to T3; possible intrahepatic hypothyroidism despite normal TSH	Sinha et al. [1], Chng et al. [2]
Metabolic dysfunction-associated steatohepatitis (MASH)	Normal or mildly increased	Normal	Normal to decreased	Low FT3 associated with inflammation and fibrosis severity	Chng et al. [2], Manka et al. [16]
Compensated cirrhosis	Normal	Normal to decreased	Decreased	Early nonthyroidal illness pattern; FT3 reduction correlates with disease severity	De Groot [29], Warner and Beckett [31]
Decompensated cirrhosis	Normal to decreased	Normal to decreased	Markedly decreased	Classic low T3 syndrome; prognostic significance for mortality	Manka et al. [16], De Groot [29]
Acute liver failure	Decreased or normal	Decreased	Decreased	Severe nonthyroidal illness syndrome due to cytokine-mediated suppression	Warner and Beckett [31]
Cholestatic liver disease	Normal	Normal to increased	Normal or decreased	Increased thyroid hormone–binding proteins may alter total hormone levels; free hormones often preserved	Khemichian and Fong [26]
Hyperthyroidism-related liver dysfunction	Decreased	Increased	Increased	Thyrotoxicosis-related hepatocellular or cholestatic injury; reversible with treatment	Khemichian and Fong [26]
Hypothyroidism-associated liver dysfunction	Increased	Decreased or normal	Decreased or normal	Dyslipidemia and steatosis; mild transaminase elevation possible	Sinha et al. [1], Taylor et al. [17]
Nonthyroidal illness syndrome (any severe liver disease)	Normal or decreased	Normal or decreased	Decreased	Adaptive response to illness; treatment generally not indicated	De Groot [29]

NTIS in liver disease

NTIS, also referred to as sick euthyroid syndrome, encompasses a spectrum of alterations in circulating TH concentrations that arise during severe systemic illness without intrinsic thyroid disease. The characteristic biochemical profile includes reduced T3 levels, normal or decreased T4 concentrations, elevated reverse T3, and TSH values that are typically low, normal, or only mildly increased [38].

The pathophysiology of NTIS is complex and involves coordinated alterations across multiple organs, including liver. Changes occur at several levels of TH signaling, including circulating transport proteins, membrane transporters, intracellular deiodinase activity, and TR expression. In chronic and critical illness, hepatic synthesis of TH-binding proteins such as TBG, transthyretin, and albumin is reduced, leading to lower total circulating T4 concentrations. In addition, qualitative changes in binding affinity of these proteins further modify the relationship between free and total hormone levels during both acute and prolonged illness [39].

Impaired hepatic handling of THs contributes further to reduced hormone availability. Accumulation of bilirubin and non-esterified fatty acids in liver disease interferes with hepatic uptake of T4 and limits its conversion to T3 [40]. Alterations in TH transporter expression also appear to be illness-stage dependent [41]. MCT8 expression is increased during acute illness, potentially representing a compensatory mechanism aimed at preserving intrahepatic T3 availability when circulating levels decline. In contrast, prolonged critical illness is associated with suppression of MCT8 expression, a change that may contribute to persistent low T3 states and adverse clinical outcomes [42].

Deiodinase activity within the liver is profoundly affected during NTIS. Expression of DIO1, the enzyme responsible for T4 activation, is markedly reduced in chronic illness, limiting local and systemic T3 generation. In contrast, DIO3, which inactivates THs, shows dynamic regulation. Its expression tends to decrease during acute illness but increases during prolonged or chronic disease states, likely reflecting adaptive shifts in hepatic energy requirements over the course of illness [43,44].

Alterations in TR expression further modulate hepatic TH action. Experimental models demonstrate that acute illness reduces hepatic expression of both TR alpha and beta, whereas chronic inflammatory states may exert less consistent effects [45,46]. In human studies, liver biopsies from patients with advanced CLD have shown increased TR expression despite low circulating TH levels. Interestingly, expression of receptor isoforms and downstream TH-responsive genes often remain relatively stable, suggesting the presence of compensatory mechanisms that preserve intracellular TH signaling [47].

Collectively, these observations indicate that despite substantial disturbances in circulating TH concentrations, the liver engages multiple adaptive responses involving transporters, deiodinases, and receptors to maintain a relative euthyroid state at the tissue level. NTIS in liver disease therefore reflects a dynamic and stage-dependent endocrine adaptation rather than simple TH deficiency, with important implications for prognosis and therapeutic decision-making [48].

Therapeutic implications and emerging treatments

THs exert direct and indirect effects on hepatic lipid and cholesterol metabolism through regulation of LDL receptor expression, fatty acid oxidation, and lipogenesis. In hypothyroidism, dyslipidemia is characterized not only by elevated cholesterol levels but also by qualitative changes in lipoprotein particles, including increased small dense LDL, which may contribute to heightened cardiometabolic risk [49,50]. Restoration of euthyroidism with LT4 improves lipid profiles in hypothyroid patients; however, iatrogenic thyrotoxicosis related to treatment has been associated with adverse cardiovascular and skeletal outcomes, underscoring the importance of cautious dose titration [51].

Preclinical studies have demonstrated that T3 and TRβ-selective agonists robustly reduce hepatic steatosis, inflammation, and fibrosis in experimental models of metabolic liver disease. Activation of hepatic TRβ signaling suppresses lipogenesis while enhancing mitochondrial fatty acid oxidation, resulting in marked reductions in liver fat content [1,9,52]. TRβ has been identified as the dominant receptor isoform mediating hepatocyte metabolic effects and proliferation, providing a strong rationale for liver-selective therapeutic targeting [3]. These effects are further supported by mechanistic studies demonstrating stimulation of lipophagy, adenosine monophosphate-activated protein kinase (AMPK) signaling, mitochondrial biogenesis, and autophagy, all of which contribute to improved hepatic lipid handling and reduced lipotoxicity.

To harness hepatic benefits while minimizing off-target thyrotoxic effects mediated through TRα in the heart, bone, and skeletal muscle, several TRβ-specific thyromimetics have been developed. Early agents such as sobetirome (GC-1) and eprotirome were designed to retain T3-like affinity for TRβ with reduced TRα activity and were initially evaluated for hypercholesterolemia. Clinical studies demonstrated substantial reductions in LDL-C, apolipoprotein B, triglycerides, and lipoprotein, with minimal effects on TSH and limited extrahepatic toxicity. However, subsequent trials raised safety concerns, including cartilage toxicity and elevations in liver enzymes, which restricted further development of first-generation compounds [53,54].

Renewed interest in this therapeutic class has emerged alongside the rising global burden of MASLD. Several next-generation liver-directed TRβ agonists, including VK-2809, resmetirom (MGL-3196), TG68, HSK31679, and other investigational compounds, have demonstrated favorable metabolic and hepatic effects in preclinical and early clinical studies [55-58]. VK-2809, a liver-activated prodrug converted by hepatic cytochrome enzymes, reduces intrahepatic lipid content, enhances β-oxidation, and promotes autophagy and mitochondrial activity in experimental models. Clinical evaluation in biopsy-confirmed MASH has shown reductions in hepatic fat, although long-term histological outcomes are still being evaluated [59].

Resmetirom, a liver-selective TRβ agonist with preferential hepatic uptake, represents a major translational advance. In phase II and III trials, it significantly reduced intrahepatic lipid content, improved atherogenic lipid profiles, and achieved meaningful histological endpoints, including MASH resolution and fibrosis improvement [10,60,61]. Its metabolic actions appear to involve activation of TRβ-regulated transcriptional pathways, increased oxygen consumption, stimulation of AMPK signaling, and modulation of inflammatory and fibrogenic pathways, including nuclear factor kappa-light-chain-enhancer of activated B cells (NF-κB) and transforming growth factor β-suppressor of mother against decapentaplegic (TGF-β-SMAD) signaling [62].

Importantly, in 2024, resmetirom became the first thyroid hormone receptor-β agonist to receive regulatory approval from the U.S. Food and Drug Administration for the treatment of adults with non-cirrhotic metabolic dysfunction-associated steatohepatitis (MASH) with moderate-to-advanced liver fibrosis (stages F2-F3), to be used alongside lifestyle modification. This milestone marks a significant shift toward targeted endocrine-metabolic therapy for steatotic liver disease and underscores the translational relevance of thyroid hormone signaling in hepatic pathophysiology [10].

In parallel, endogenous TH metabolites such as 3,5-diiodo-L-thyronine have emerged as potential anti-steatotic agents that improve insulin sensitivity and hepatic lipid metabolism without overt thyrotoxic effects [63-65]. Additionally, innovative strategies including nanoparticle-based delivery systems and hybrid molecules integrating TH signaling with other metabolic pathways, such as glucagon, have demonstrated enhanced liver selectivity and metabolic efficacy in experimental models [66,67].

Beyond lipid metabolism, thyromimetics may exert broader effects on inflammation and fibrosis through modulation of macrophage activation, hepatic stellate cell signaling, and cellular stress responses [9]. TH-mediated stimulation of autophagy, reduction of endoplasmic reticulum stress, and suppression of inflammasome activation may contribute to hepatoprotection, although the precise contribution of direct anti-inflammatory versus lipid-lowering mechanisms remains incompletely understood. Differential activation of TRα and TRβ across hepatic and extrahepatic tissues further adds complexity, as TRα signaling may influence stellate-cell plasticity and fibrogenesis, whereas TRβ-selective agents primarily target metabolic pathways within hepatocytes [9,66].

Emerging therapeutic paradigms increasingly favor precision-based and combination approaches that integrate TH signaling with other metabolic pathways. Collectively, these advances highlight the considerable promise of TH-based and TRβ-selective therapies in MASLD and MASH. However, long-term safety, durability of histological benefits, and the balance between receptor-selective metabolic efficacy and potential extrahepatic effects require continued investigation before widespread clinical adoption [66,67]. Table 2 summarizes major TRβ-selective thyromimetics, their mechanisms, clinical development stages, and key hepatic and metabolic outcomes. It highlights the growing therapeutic potential of these agents in MASLD/MASH, with resmetirom having the strongest clinical evidence, while other compounds remain under active investigation with ongoing evaluation of efficacy and safety.

**Table 2:** Thyroid hormone receptor-β-selective agonists and emerging therapies in metabolic liver disease TRβ: thyroid hormone receptor beta; TH: thyroid hormone; MASH: metabolic dysfunction-associated steatohepatitis; MASLD: metabolic dysfunction-associated steatotic liver disease; LDL-C: low-density lipoprotein cholesterol; ApoB: apolipoprotein B; TAG: triglycerides; GI: gastrointestinal; CV: cardiovascular; β-oxidation: beta-oxidation; NAFLD: nonalcoholic fatty liver disease; T2: 3,5-diiodo-L-thyronine; AMPK: AMP-activated protein kinase.

Drug / Compound	Mechanism of action	Key hepatic/metabolic effects	Clinical evidence	Safety concerns	References
Resmetirom (MGL-3196)	Liver-targeted selective TRβ agonist; enhances hepatic TH signaling, lipid metabolism, mitochondrial function	Reduces hepatic fat, improves lipid profile, promotes MASH resolution and fibrosis improvement	Phase II and III trials demonstrated reduction in liver fat and significant histological improvement in MASH	Mild GI symptoms, lipid changes; long-term CV and skeletal safety under evaluation	Harrison et al. [10], Harrison et al. [60], Wang et al. [62], Hovingh et al. [68], Hones et al. [69]
VK2809 (MB07811)	Prodrug converted in liver; selective TRβ activation; enhances β-oxidation and lipid clearance	Reduces steatosis and intrahepatic lipid accumulation in preclinical and early clinical studies	Phase IIb VOYAGE trial completed; ongoing evaluation in MASH	Limited long-term safety data	Erion et al. [55], Cable et al. [56], Zhou et al. [59]
Sobetirome (GC-1)	TRβ-selective thyromimetic; stimulates cholesterol metabolism and hepatic lipid utilization	Decreases LDL-C and hepatic lipid accumulation in experimental models	Early-phase clinical studies; not advanced due to development limitations	Long-term efficacy and safety not established	Perra et al. [52], Johansson et al. [70], Lammel Lindemann and Webb [71]
Eprotirome (KB2115)	TRβ-selective agonist with lipid-lowering action	Reduces LDL-C, ApoB, triglycerides	Phase II lipid studies positive; Phase III halted due to cartilage toxicity and liver enzyme elevations	Hepatotoxicity signals; cartilage toxicity in animals	Ladenson et al. [53], Sjouke et al. [54]
TG68	Novel TRβ agonist targeting hepatic lipid metabolism	Reduces steatosis and improves metabolic parameters in preclinical NAFLD models	Preclinical stage	Clinical safety unknown	Caddeo et al. [57], Caddeo et al. [72]
HSK31679 / ALG-055009 / emerging TRβ agonists	Next-generation selective thyromimetics with improved liver selectivity	Potential reduction in hepatic fat, inflammation, fibrosis	Early clinical development	Data evolving	Hu et al [58], Luong et al. [73]
3,5-diiodo-L-thyronine (T2)	Thyroid hormone metabolite enhancing mitochondrial oxidation and lipid catabolism	Anti-steatotic effects, improved insulin sensitivity in experimental models	Preclinical	Human safety and dosing unclear	de Lange et al. [63], Iannucci et al. [64], Sane et al. [65]

The quality and consistency of evidence across different aspects of the thyroid-liver axis remain heterogeneous. While epidemiological studies, mechanistic investigations, and phase III clinical trials provide strong support for the role of thyroid dysfunction and TRβ-selective agonists in metabolic liver disease, several areas including thyroid hormone sensitivity indices, long-term safety of thyromimetics, and liver-directed levothyroxine therapy still require further validation. Table 3 summarizes the current strength of evidence supporting major mechanistic, diagnostic, prognostic, and therapeutic concepts discussed in this review. A summary of the key studies included in this review is provided in Appendix 1.

**Table 3:** Strength of evidence supporting major concepts in the thyroid-liver axis FT3: free triiodothyronine; TSH: thyroid-stimulating hormone; TH: thyroid hormone; TFT: thyroid function test; LT4: levothyroxine; NTIS: nonthyroidal illness syndrome; TRβ: thyroid hormone receptor beta; MASLD: metabolic dysfunction-associated steatotic liver disease; MASH: metabolic dysfunction-associated steatohepatitis.

Major concept	Evidence strength	Type of supporting evidence	Key references	Clinical implication
Hypothyroidism contributes to hepatic steatosis and MASLD progression	Strong	Epidemiological studies, mechanistic studies, meta-analyses	Bano et al. [4], Sinha et al. [1], Taylor et al. [17]	Thyroid dysfunction may contribute to metabolic liver disease and should be considered during evaluation of MASLD
Reduced intrahepatic thyroid hormone signaling contributes to steatosis and fibrosis	Moderate to strong	Experimental models, translational liver studies	Zhou et al. [9], Manka et al. [16], Chng et al. [2]	Supports the concept of intrahepatic hypothyroidism and impaired tissue-level TH action
Low FT3 levels correlate with cirrhosis severity and mortality	Strong	Observational cohort studies and prognostic analyses	Hartl et al. [30], Nardin et al. [36], Bao et al. [37]	FT3 may serve as a prognostic biomarker in advanced liver disease
TSH alone may inadequately reflect tissue-level thyroid hormone action in liver disease	Moderate	Clinical studies evaluating TH sensitivity and NTIS	Fliers et al. [8], Laclaustra et al. [33], Chng et al. [2]	Interpretation of TFTs in liver disease requires caution
Levothyroxine therapy may improve hepatic steatosis in selected hypothyroid patients	Limited to moderate	Small interventional studies and post hoc analyses	Zhou et al. [9], Stott et al. [22]	Evidence remains insufficient for routine liver-directed LT4 therapy
NTIS represents an adaptive endocrine response rather than true hypothyroidism	Moderate to strong	Critical illness endocrine studies and mechanistic data	Van den Berghe [7], Fliers et al. [8], Boonen and Van den Berghe [34]	Routine thyroid hormone replacement is generally not recommended in NTIS
TRβ-selective agonists improve hepatic steatosis and metabolic parameters	Strong	Phase II and III clinical trials, preclinical studies	Harrison et al. [10], Harrison et al. [60], Perra et al. [52]	Represents a promising therapeutic strategy for MASH
Long-term cardiovascular and skeletal safety of thyromimetics remains uncertain	Limited	Extension studies and safety analyses	Ladenson et al. [53], Sjouke et al. [54], Ochani et al. [51]	Long-term safety monitoring remains necessary
Resmetirom improves histological outcomes in MASH	Strong	Phase III randomized controlled trial	Harrison et al. [10], Petta et al. [11]	First approved TRβ agonist for MASH treatment
Thyroid hormone sensitivity indices may improve risk stratification in liver disease	Emerging/limited	Observational and exploratory studies	Laclaustra et al. [33], Bao et al. [37]	Requires prospective validation before routine clinical use

## Conclusions

The thyroid-liver axis constitutes a critical endocrine-metabolic interface in which THs regulate hepatic lipid metabolism, mitochondrial function, and inflammatory pathways, while liver disease profoundly alters TH transport, metabolism, and tissue sensitivity. Evidence across the spectrum of liver disease indicates that hypothyroidism contributes to metabolic liver dysfunction, hyperthyroidism may cause liver enzyme derangements, NTIS reflects disease severity, and impaired peripheral TH sensitivity carries prognostic significance beyond conventional thyroid function tests.

Clinically, recognition of thyroid-liver interactions may improve the interpretation of thyroid function abnormalities and support risk stratification in metabolic liver disease. Although LT4 therapy may benefit hypothyroid patients with MASLD, its role in euthyroid liver disease is limited by safety concerns. In contrast, liver-targeted TRβ-selective agonists and emerging combination metabolic therapies offer promising therapeutic potential with improved specificity. Further mechanistic studies and long-term clinical trials are needed to validate TH sensitivity markers, optimize patient selection, and establish the long-term cardiovascular and skeletal safety of thyroid hormone-based therapies before their routine integration into clinical practice.

## Appendices

Appendix 1

**Table 4:** Summary of key studies included in the review ACLF: acute-on-chronic liver failure; FT3: free triiodothyronine; FDA: United States Food and Drug Administration; ICU: intensive care unit; LDL-C: low-density lipoprotein cholesterol; MASLD: metabolic dysfunction-associated steatotic liver disease; MASH: metabolic dysfunction-associated steatohepatitis; NAFLD: nonalcoholic fatty liver disease; NASH: nonalcoholic steatohepatitis; NTIS: nonthyroidal illness syndrome; TH: thyroid hormone; TRα: thyroid hormone receptor alpha; TRβ: thyroid hormone receptor beta; TSH: thyroid-stimulating hormone.

Study	Study design	Population / Model	Main findings	Clinical relevance
Bano et al., 2016 [4]	Prospective population-based cohort study	Participants from the Rotterdam Study	Higher TSH levels and low-normal thyroid function were associated with increased risk of NAFLD	Supports association between hypothyroidism and MASLD
Sinha et al., 2018 [1]	Narrative mechanistic review	Experimental and translational evidence	TH regulates hepatic lipid metabolism, β-oxidation, and cholesterol homeostasis	Establishes mechanistic basis of thyroid-liver interactions
Zhou et al., 2022 [9]	Experimental animal study	Dietary mouse model of NASH	Thyroid hormone therapy reduced steatosis, inflammation, and fibrosis	Supports therapeutic role of TH signaling in MASH
Harrison et al., 2019 [60]	Phase II randomized controlled trial	Patients with biopsy-confirmed NASH	Resmetirom significantly reduced hepatic fat content and improved lipid profile	Early clinical validation of TRβ agonist therapy
Harrison et al., 2024 [10]	Phase III randomized controlled trial (MAESTRO-NASH)	Patients with MASH and liver fibrosis	Resmetirom improved MASH resolution and fibrosis endpoints	Landmark trial supporting FDA approval of resmetirom
Petta et al., 2024 [11]	Contemporary review	Clinical and translational studies	Summarized emerging role of resmetirom in MASH treatment	Highlights translational significance of TRβ agonists
Hartl et al., 2024 [30]	Observational cohort study	Patients with cirrhosis	Low FT3 levels correlated with systemic inflammation, ACLF risk, and mortality	Demonstrates prognostic role of FT3 in advanced liver disease
Nardin et al., 2024 [36]	Observational prognostic study	Patients with acute decompensated cirrhosis	Altered thyroid hormone profile associated with worse prognosis	Supports prognostic utility of thyroid indices
Laclaustra et al., 2019 [33]	Population-based observational study	Adults with metabolic syndrome and diabetes risk	Impaired thyroid hormone sensitivity associated with diabetes and metabolic syndrome	Supports emerging concept of TH resistance/sensitivity
Feng et al., 2020 [35]	Clinical observational study	Patients with liver failure	NTIS was associated with severity of liver failure and adverse outcomes	Supports clinical significance of NTIS in liver disease
Perra et al., 2008 [52]	Experimental animal study	Rat model of NAFLD	TRβ agonist GC-1 reversed hepatic steatosis	Foundational evidence for thyromimetic therapy
Erion et al., 2007 [55]	Preclinical pharmacological study	Experimental liver-targeted TRβ agonist models	Liver-selective TRβ agonists improved lipid metabolism and reduced steatosis	Supported development of liver-directed thyromimetics
Cable et al., 2009 [56]	Experimental animal study	Mouse and rat steatosis models	Liver-targeted TRβ agonist reduced hepatic fat accumulation	Reinforced therapeutic role of selective TH signaling
Ladenson et al., 2010 [53]	Randomized clinical trial	Statin-treated dyslipidemia patients	Eprotirome reduced LDL-C and triglycerides	Demonstrated metabolic efficacy of TRβ agonists
Sjouke et al., 2014 [54]	Phase III randomized trial	Familial hypercholesterolemia patients	Eprotirome improved lipids but raised cartilage and hepatic safety concerns	Highlighted safety limitations of early thyromimetics
Manka et al., 2024 [16]	Mechanistic translational study	Hepatic stellate cells and fibrosis models	TRα signaling modulated hepatic fibrogenesis	Expanded understanding of thyroid receptor biology in fibrosis
Bao et al., 2026 [37]	Systematic review and meta-analysis	Patients with liver failure and thyroid dysfunction	Thyroid dysfunction associated with poor prognosis in liver failure	Supports prognostic significance of thyroid abnormalities
Van den Berghe, 2014 [7]	Review of critical illness endocrinology	ICU and critical illness studies	NTIS reflects adaptive endocrine responses during severe illness	Supports cautious interpretation of thyroid function tests in illness
Fliers et al., 2015 [8]	Review article	Critically ill patients	Described mechanisms and interpretation challenges of NTIS	Provides framework for thyroid assessment in severe liver disease
